# Cervical cancer and precancerous cervical lesions detected using visual inspection with acetic acid at Livingstone Teaching Hospital

**DOI:** 10.11604/pamj.2021.40.235.32300

**Published:** 2021-12-17

**Authors:** Valerie Hayumbu, Joy Hangoma, Benson Malambo Hamooya, Malan Malumani, Sepiso Kenias Masenga

**Affiliations:** 1 Mulungushi University, School of Medicine and Health Sciences, Hand Research Group, Livingstone, Zambia

**Keywords:** Visual inspection with acetic acid (VIA), cervical cancer, HIV

## Abstract

**Introduction:**

cervical cancer (CaCx) is the second most common malignancy in women world-wide. Precancer screening aided by visual inspection with acetic acid (VIA) is an early diagnosis method used to detect the lesions that are high indicators of cancer in women. cervical cancer is more prevalent in the developing world affecting mainly women in the reproductive age group and is the commonest cancer among Zambian women. Therefore, the study aimed to determine the prevalence and factors associated with a positive VIA at Livingstone Teaching Hospital (LTH).

**Methods:**

this was a cross-sectional study conducted at LTH among 329 women from Livingstone district aged 18 and above, who were coming for routine cervical cancer screening using VIA between 2019 and 2020. Demographic and clinical data were collected from the CaCx clinic. A positive VIA (precancerous cervical lesions) and cervical cancer were the primary and secondary outcome variables. A positive VIA was defined by presence of a dense ulcerative acetowhite area in the transformation zone of the cervix. Cervical cancer diagnosis was defined by presence of cancerous cells on histological examination by a qualified pathologist. Data were analyzed using Statistical package for social sciences (SPSS) version 22.0. Chi-square test, Mann-Whitney and logistic regression were the statistical methods used.

**Results:**

the participants had a median (interquartile range) age of 37 (29, 44) years. Prevalence of CaCx and positive VIA were 6% (95% confidence interval (CI) 4, 9) and 19% (95% CI: 15, 24) respectively. At multivariable analysis, the factors associated with a positive VIA were alcohol consumption [odds ratio (OR) 0.30 (95% CI: 0.12, 0.74)] and HIV infection [OR 0.37 (95% CI: 0.19, 0.70)].

**Conclusion:**

the study showed that precancerous cervical lesions are common among our study participants and it was influenced by alcohol consumption and HIV status. There is therefore need to enhance the screening programs using VIA in order to identify cancerous lesions at an early stage for early intervention in resource limited settings.

## Introduction

Cervical cancer (CaCx) is the most common cause of death and is the second most common malignancy in women world-wide [1]. The incidence of CaCx is rapidly increasing especially in low- and middle-income countries (LMICs) like Zambia [2,3]. In the developed world, the disease has minimal impact and is uncommon owing to the cytological methods of screening, education and access to good medical care which has greatly reduced the morbidity and mortality of CaCx [4]. The rationale for screening for CaCx is based on evidence of infection with human papillomavirus (HPV) and/or the presence of cervical epithelial changes [3,5]. The World Health Organization (WHO) recommends use of visual inspection methods such as visual inspection with acetic acid (VIA) to screen for CaCx in poor resource countries [4]. Visual inspection with acetic acid is a simple, safe, feasible, accurate, acceptable and easily accessible method for pre-CaCx screening [6]. A positive VIA test is a risk factor for CaCx [7]. Studies that report the factors associated with VIA positive result are scarce in sub-Saharan Africa (SSA). We conducted a study to determine the prevalence and factors associated with a positive VIA test at Livingstone Teaching Hospital (LTH).

## Methods

**Study design and setting:** we conducted a cross sectional study at LTH cervical cancer clinic between January 2019 to December 2020. Livingstone teaching hospital was selected as it is the largest referral hospital in Southern Province of Zambia, and the only hospital where cervical cancer screening is conducted in Southern Province.

**Eligibility criteria:** the study included all adult females above 18 years that was screened for cervical cancer between January 2019 and December 2020. Women who had been screened but had missing data on VIA result and results for cervical cancer diagnosis were excluded from the study.

**Selection of participants:** we used systematic random sampling to select participants by dividing the population size (N) or total number of women screened with the sample size (n) to compute the sampling interval (k). We then selected every kth participant. So, the estimated total number (N) of women screened between January 2019 to December 2020 was 2917. We divided 2917 by our maximal sample size (n=385) to get the sampling interval 7.5. Hence, we recruited every seventh woman using the clinic´s register. We had to recall the participants that were selected and offered them to participate in the study. Participants were only enrolled after signing a written consent. A total sample size of 329 was arrived at after excluding 56 participants due to missing information on VIA positivity status.

**Sample size estimation:** with anticipated prevalence of 5.9% from a previous study [8], we required a minimal sample size of 86 and a maximum sample size (50% expected prevalence) of 385 at 95% confidence interval with ±5% confidence limits using OpenEpi info software.

**Data collection:** we used an interviewer structured questionnaire to collect sociodemographic and clinical data from the participants and additional data from their medical files.

**Study variables:** a positive VIA (precancerous cervical lesion) and cervical cancer were the primary and secondary outcome variables, respectively. The screening test and diagnostic procedures for cervical cancer were performed by a trained medical doctor. A sterile Cusco's self-retaining vaginal speculum was inserted followed by application of freshly prepared 5% acetic acid to the cervix. The cervix was visualized under illuminated light from a halogen lamp. A positive test was indicated when a dense acetowhite area with regular margins appeared attached to the squamo columnar junction in the transformation zone [9]. Precancerous lesions have higher protein content when compared to normal epithelium, hence, appear white (acetowhite) with acetic acid. Absence of ulcerative lesions indicated a negative test. All VIA positive participants were treated with cryotherapy by the attending medical doctor. Cervical punch biopsies were obtained for lesions covering more than 75% of the cervical epithelium with evidence of advanced cervical intraepithelial neoplasia (CIN), and sent to the histopathology lab for CaCx diagnosis. The sociodemographic and clinical independent variables in this study were age, marital status, employment status, highest educational level, smoking status, alcohol consumption, number of full-term pregnancies, history of tuberculosis, type of family planning used, family history of cervical cancer, cervical cancer diagnosis or status, cumulative number of cervical cancer screening done, HIV status, history of sexually transmitted disease, age at first coitus and use of family planning. Except for age and number of screenings, all variables were categorical. Data on the use of family planning, history of tuberculosis, cervical cancer diagnosis, HIV status and cumulative number of cervical cancer screening was provided by the participant but verified in the medical files.

**Statistical analysis:** the collected data was entered in Microsoft Excel and then exported to Statistical package for social sciences (SPSS) version 22. To test whether the data in the study population was normally distributed, Shapiro Wilks test was used. To describe our data, we used descriptive statistics such as medians with interquartile range for continuous data, frequencies and percentages for categorical data. The outcome variable was a positive VIA. The independent variables included age, gender, education status, smoking status, full term pregnancy, employment status, alcohol consumption, marital status, history of tuberculosis, cervical cancer, family history of cervical cancer, number of cervical cancer screening done, history of sexually transmitted infections, age at first coitus, family planning use, type of family planning used and HIV status. To compare the relationship between a positive VIA and related categorical variables, the chi-square test was used. Mann Whitney test was used to compare the statistical difference between two medians. Univariate and multivariable logistic regression was used to ascertain the factors associated with a positive VIA and control for confounding. In the multivariable model, we included the variables that were statistically significant at univariate analysis and those that were of clinical significance in contributing to the outcome and supported by literature. P-value of less than 0.05 was considered to be statistically significant at 95% confidence Interval.

**Ethical considerations:** ethical approval was obtained from the Mulungushi University School of Medicine and Health Sciences Research Ethics Committee (IRB: 00012281 FWA: 0002888). Permission to conduct the study was granted by Livingstone Teaching Hospital Administration. Data collected was de-identified and used for research purposes only.

## Results

**General characteristics:** the study comprised 329 female participants with median age (interquartile range) 37 years (29, 44). The prevalence of cervical cancer was 6% (95% confidence interval (CI) 4, 9) and 19% (95% CI: 15, 24) for VIA positivity. 63% (208/329) were HIV positive. Employment status, alcohol consumption, marital status, cervical cancer and HIV were associated with a positive VIA, p<0.05, Table 1.

**Table 1:** factors associated with visual inspection with acetic acid status

Variable	VIA result		
	Positive, n (%)	Negative, n (%)	p-value
Median age (IQR)37(29, 44)	37 (29, 43)	37 (29, 43)	0.178
**Marital status**			
Single	7 (11.1)	50 (18.8)	0.043
Married	50 (79.4)	167 (62.8)	
Widowed or divorced or separated	6 (9.5)	49 (18.4)	
**Employment status**			
Informal	33 (52.4)	80 (30.1)	0.002
Formal employment	11 (17.5)	88 (33.1)	
Unemployed	19 (30.2)	98 (36.8)	
**Highest educational Level**			
Primary school	13 (20.6)	38 (14.3)	0.39
Secondary school	29 (46.0)	122 (45.9)	
Tertiary	21 (33.3)	106 (39.8)	
**Smoking status**			
Smoker	0 (0)	11 (4.1)	0.13
Non smoker	63 (100)	255 (95.9)	
**Alcohol consumption**			
Yes	7 (11.1)	91 (34.2)	<0.001
No	56 (88.9)	175 (65.8)	
**Full term Pregnancy**			
Yes	56 (88.9)	229 (86.1)	0.56
No	7 (11.1)	37 (13.9)	
**Number of Full-term Pregnancies**			
None	6 (9.7)	36 (13.5)	0.61
One	6 (9.7)	31 (11.7)	
Multiple	50 (80.6)	199 (74.8)	
**History of Tuberculosis**			
Yes	5 (7.9)	21 (8.0)	0.98
No	58 (92.1)	241 (92.0)	
Age at first coitus	37 (42, 36)	37 (29, 43)	0.73
**Type of family planning used**			
None	16 (25.4)	80 (30.1)	0.47
Hormonal	38 (60.3)	138 (51.9)	
Barrier method	9 (14.3)	48 (18.0)	
**Family history of cervical cancer**			
Yes	0 (0.0)	3 (3.4)	1.00
No	6 (100)	84 (96.6)	
**Cervical cancer status**			
Negative	43 (68.3)	266 (100.0)	<0.001
Positive	20 (31.7)	0 (0.0)	
**Number of screenings done**, median (IQR)	1.0 (1.0, 1.0)	1.0 (1.0, 1.0)	0.12
**HIV status**			
HIV positive*	27 (43.5)	181 (68.0)	<0.001
HIV negative	35 (56.5)	85 (32.0)	
**Ever had STIs**			
Yes	0 (0.0)	13 (14.4)	1.00
No	6 (100.0)	77 (85.6)	
**Family planning use**			
Yes	47 (74.6)	187 (70.3)	0.50
No	16 (25.4)	79 (29.7)	

* All HIV positive participants were virally suppressed; visual inspection with acetic acid( VIA); HIV, human immunodeficiency virus; sexually transmitted infections(STIs); interquartile range (IQR)

**Correlates of a positive VIA:** we performed a multivariable logistic regression to determine the contribution of each variable towards a positive VIA test, Table 2. At multivariable analysis, the factors significantly associated with a positive VIA test were alcohol consumption in which participants who consumed alcohol had 70% (OR 0.30; 95%CI 0.12, 0.74; p=0.009) lower odds of having a positive VIA, and HIV positive individuals had 63% (OR 0.37; 95%CI 0.19, 0.70; p=0.002) lower odds of having a positive VIA. The other variables were comparable.

**Table 2:** factors associated with a positive VIA in logistic regression

Variable	Multivariable analysis adjusted odds ratio (95% CI)	p-value
Participant's age	1.02 (0.99 - 1.06)	0.14
**Age at first coitus**	0.87 (0.75-1.01)	0.07
**Marital status**		
Single	1	
Married	2.16 (0.66 - 7.02)	0.19
Widowed or divorced or separated	0.85 (0.19 - 3.82)	0.83
**Full term pregnancies**		
None	1	
One	0.41 (0.10 - 1.68)	0.21
Multiple	0.32 (0.08 - 1.23)	0.09
**Alcohol consumption**		
No	1	
Yes	0.30 (0.12 - 0.74)	0.009
**History of tuberculosis**		
No	1	
Yes	0.60 (0.19 - 1.81)	0.36
**HIV status**		
Negative	1	
Positive	0.37 (0.19 - 0.70)	0.002
**Use of family planning**		
No	1	
Yes	1.50 (0.71 - 3.20)	0.28

visual inspection with acetic acid (VIA); human immunodeficiency virus (HIV); confidence interval(CI)

## Discussion

This study was aimed at determining the prevalence and factors associated with a positive VIA and the burden of CaCx. We found that the prevalence of a positive VIA and cervical cancer were 19% and 6%, respectively. At multivariable analysis only alcohol consumption and HIV status remained significantly negatively associated with VIA positivity. The prevalence of a positive VIA based on what others have reported ranges from 10-43% [10-12]. Cervical VIA is recommended for low resource settings [13], it is cheap and its performance is comparable to the Papanicolaou test [14]. Based on the results of this study, both alcohol and HIV status were protective against a positive VIA. This is contrary to previous reports where they found that a positive VIA was more likely among HIV positive women [15-17]. Although HIV viral suppression reduces the risk of cervical cancer and precancerous cervical lesions [18], it was beyond the scope of this study to ascertain why the odds for a positive VIA were lower in HIV positive women compared to the HIV negative women. A follow up study is required to validate these findings. According to this study, alcohol consumption was significantly negatively associated with VIA positivity. Women with a history of alcohol consumption were less likely to have a positive VIA result as compared to those who never consumed alcohol, the reason is unknown. There are no studies conducted in Africa known to us that report the effects of alcohol on VIA positivity. However, previous studies (reviews) from elsewhere have not found strong evidence of a possible relationship between alcohol intake and cervical cancer [19].

**Strengths and limitations of the study:** this study utilized systematic random sampling to estimate the prevalence of cervical cancer and precancerous cervical lesions. This method is easy to use, reliable and eliminates the phenomenon of clustered selection and has a low probability of contaminating data. The use of multivariable logistic regression minimized confounding and since this method considers more than one factor of independent variables that influence the variability of the dependent variable, the conclusion drawn is more reliable. This study did not investigate the prevalence of HPV and its subtypes to correlate HPV and VIA. This would have provided more information on the interaction and contribution of HPV towards the outcome variable in relation to other independent variables. Since this was a single site study, these findings might not be generalizable to other populations. Larger studies with large sample size are needed to validate our findings. The study is limited by confounding from measured and unmeasured confounders. In addition, we lacked the statistical power to assess the association between outcome and some exposures like smoking where we had some cells with zero observations. A possibility of reverse causation is also likely, hence the need for further investigation.

## Conclusion

Our study identified a high percentage of precancerous cervical lesions (19%) and low percentage of cervical cancer (6%) among women attending routine cervical cancer screening at Livingstone Teaching Hospital. Although we found that HIV infection and alcohol consumption were negatively associated with a positive VIA, a follow up study is needed to validate this. VIA is a cheap and efficient method to detect high-level cervical cell dysplasia and we recommend this for low-income countries.

### What is known about this topic

- *A positive VIA is a risk factor for CaCx;*
- *HIV infection is positively associated with a positive VIA*.

### What this study adds

- *HIV infection and alcohol consumption were negatively associated with a positive VIA;*
- *The prevalence of a positive VIA and CaCx is 19% and 6%, respectively*.
